# A Novel Nomogram Based on Quantitative MRI and Clinical Features for the Prediction of Neonatal Intracranial Hypertension

**DOI:** 10.3390/children10101582

**Published:** 2023-09-22

**Authors:** Yan Qin, Yang Liu, Chuanding Cao, Lirong Ouyang, Ying Ding, Dongcui Wang, Mengqiu Zheng, Zhengchang Liao, Shaojie Yue, Weihua Liao

**Affiliations:** 1 Department of Radiology, Xiangya Hospital, Central South University, 87 Xiangya Road, Changsha 410008, China; 2 National Clinical Research Center for Geriatric Disorders, Xiangya Hospital, Central South University, 87 Xiangya Road, Changsha 410008, China; 3 Department of Pediatrics, Xiangya Hospital, Central South University, 87 Xiangya Road, Changsha 410008, Chinashaojieyue@163.com (S.Y.)

**Keywords:** neonate, intracranial hypertension, magnetic resonance imaging, prediction, volumetric segmentation

## Abstract

Intracranial hypertension (ICH) is a serious threat to the health of neonates. However, early and accurate diagnosis of neonatal intracranial hypertension remains a major challenge in clinical practice. In this study, a predictive model based on quantitative magnetic resonance imaging (MRI) data and clinical parameters was developed to identify neonates with a high risk of ICH. Newborns who were suspected of having intracranial lesions were included in our study. We utilized quantitative MRI to obtain the volumetric data of gray matter, white matter, and cerebrospinal fluid. After the MRI examination, a lumbar puncture was performed. The nomogram was constructed by incorporating the volumetric data and clinical features by multivariable logistic regression. The performance of the nomogram was evaluated by discrimination, calibration curve, and decision curve. Clinical parameters and volumetric quantitative MRI data, including postmenstrual age (*p* = 0.06), weight (*p* = 0.02), mode of delivery (*p* = 0.01), and gray matter volume (*p* = 0.003), were included in and significantly associated with neonatal intracranial hypertension risk. The nomogram showed satisfactory discrimination, with an area under the curve of 0.761. Our results demonstrated that decision curve analysis had promising clinical utility of the nomogram. The nomogram, incorporating clinical and quantitative MRI features, provided an individualized prediction of neonatal intracranial hypertension risk and facilitated decision making guidance for the early diagnosis and treatment for neonatal ICH. External validation from studies using a larger sample size before implementation in the clinical decision-making process is needed.

## 1. Introduction

Intracranial hypertension (ICH), also known as increased intracranial pressure (ICP), is a serious threat to the health of neonates and remains a global public health concern. It is often caused by asphyxia, hypoxic-ischemic encephalopathy (HIE), birth trauma, cerebral hemorrhage, infections, and severe hyperbilirubinemia, as well as some metabolic disorders [1]. The incidences of ICH in the pediatric population varies between countries. For example, the annual pediatric incidence of ICH is approximately 0.63 per 100,000 children in the Midwest [2]. It is about 0.47 per 100,000 annually in German pediatric patients [3], while the ratio is higher in Croatia and Nova Scotia and Prince Edward Island (1.2 and 0.9, respectively) [4,5]. In China, there are no accurate reports. Neonatal ICH (NICH) can directly lead to poor neurological outcomes and quality of life. Thus, early detection of NICH is essential for decreasing the risk of poor neurological sequelae and is also beneficial for reducing the related socioeconomic burden. However, NICH is often latent and is easily ignored due to the lack of appropriate expression in neonates and the unspecific clinical symptoms. Invasive intracranial monitoring or lumbar puncture is regarded as the gold standard for assessing ICP. However, these methods have a risk of hemorrhage, infection, cerebrospinal fluid (CSF) leakage, and cerebral herniation [6], and so most parents may hesitate to agree to the use of these methods for their children.

Conventional imaging studies of MRI have shown that ICH may result in morphological changes in the ventricular system, optic nerve sheath diameter, and pituitary gland. For example, Hu [7] et al. found that intracranial pressure is associated with the tortuosity and diameter of optic nerve sheath. Specifically, the higher the intracranial pressure, the larger the diameter and the smaller the angle (or increased tortuosity) of the optic nerve sheath. Sekhon’s [8] findings are consistent with this study. They also found that optic nerve sheath diameter is strongly positively associated with intracranial pressure. In addition, their study demonstrated that optic nerve sheath diameter is a strong predictor of intracranial pressure. The morphology of the ventricular system is closely related to the type of intracranial hypertension. For idiopathic intracranial hypertension patients, the morphology of the ventricular system is usually normal. In patients with intracranial hypertension caused by hydrocephalus, the ventricular systems are often significantly dilated. Changes in pituitary morphology can also reflect intracranial pressure. The pituitary gland is generally relatively full and the hypophysial diameter is enlarged in spontaneous intracranial hypotension patients. However, patients with idiopathic intracranial hypertension showed the opposite. Yuh [9] et al. found that the ratio of pituitary gland and sellar region area decreased significantly in idiopathic intracranial hypertension patients compared with acute intracranial hypertension patients and normal controls. Pituitary gland morphology has no statistically significant change in patients with acute intracranial hypertension, which may be related to the duration and severity of elevated intracranial pressure. However, changes in these areas are usually secondary and are susceptible to subjective experience and interobserver variability. In addition, some mild ICH cases do not cause morphological changes, and thus the application of these approaches was limited. Moreover, most of these studies focused on an adult population. Hence, for neonatologists and anxious parents, an alternative, noninvasive, and accurate method for detecting NICH early and accurately is required.

In the current study, we aimed to establish a predictive model to identify neonates with a high risk of ICH. Quantitative MRI was used to provide volumetric measurements of cerebral white matter (WM), gray matter (GM), and cerebrospinal fluid (CSF). Clinical parameters that might be associated with ICP were also taken into account in the model development. Our work is expected to provide a useful and convenient tool in the diagnosis and management of ICH in neonates.

## 2. Materials and Methods

### 2.1. Participants

Neonates with suspected intracranial lesions in the neonatal intensive care unit (NICU) of our hospital were included in our study. The inclusion criteria were as follows: (1) neonates with suspected intracranial lesions, (2) who were 37 weeks ≤ gestational age at birth < 42 weeks, (3) and were ≤ 28 days old. The following neonates were excluded: those who were born prematurely or who had severe hepatorenal dysfunction, cardiac insufficiency, congenital genetic diseases, intracranial tumors, hydrocephalus, or contraindications for MRI examination. The collected clinical parameters include gender, gestational age, postmenstrual age, head circumference, mode of delivery, weight, and Apgar scores. In our study, we defined postmenstrual age as the gestational age at birth plus days of life at the time of examination. Neonates were examined while sleeping. Sedation with chloral hydrate was performed only if the neonate woke up during the examination.

All enrolled neonates underwent an MRI examination and lumbar puncture. Lumbar CSF pressure was measured before any CSF was removed, with the patient in a lateral decubitus position. Based on prior knowledge [10,11], we classified neonates with ICP > 80 mm H_2_O as the ICH group and those with ICP ≤ 80 mm H_2_O as the non-ICH group. The study was approved by the institutional ethics committee of our hospital (Approval Code: 201311417). The parents of all participants provided written informed consent to participate in the study.

### 2.2. Patients and Public Involvement

Patients or the public were not involved in the design, conduct, reporting, or dissemination plans of our research.

### 2.3. Details of MRI Acquisition

MRI data were acquired with a Siemens Prisma 3.0-T scanner (Siemens Healthcare, Erlangen, Germany) using a 64-channel head/neck receiver coil. The data for each subject consisted of a sagittal, three-dimensional, T1-weighted magnetization-prepared 2 inversion-contrast rapid gradient-echo (3D-T1 MP2RAGE) with the following parameters: TR/TE = 3600 ms/3.15 ms, flip angle = 4°, TI = 1100 ms/3000 ms, BW = 240 Hz, voxel size = 0.78 × 0.78 × 0.78, number of slices = 144 s, slice thickness = 0.78 mm. 

### 2.4. MR Image Processing

First, we converted the 3D-T1 images from digital imaging and communications in medicine format into neuroimaging informatics technology initiative format by using MRIcron software version 1.0.20190902 (http://people.cas.sc.edu/rorden/mricron, accessed on 5 March 2020). Then, automatic image segmentation and volume measurement of the newborn brain were performed using MATLAB R2013b (Math Works, Inc., Natick, MA, USA) and SPM12 (Statistical Parametric Mapping, https://www.fil.ion.ucl.ac.uk/spm/software/spm12/, accessed on 20 March 2020). 

Here, the publicly available atlas (http://brain-development.org/brain-atlases/multi-structural-neonatal-brain-atlas/, accessed on 20 March 2020) was used as the anatomical priors corresponding to the gestational ages for segmentation. First, we obtained 3 tissue probability maps (TPMs) from the atlas: GM, WM, and CSF. Second, the brain volumes were registered to the corresponding brain atlas space using Coregister in SPM12. Finally, tissue segmentation was performed by using the TPMs and the old segment algorithms of the SPM12, and then volumes of the 3 tissues were calculated by the in-house scripted MATLAB functions.

### 2.5. Model Development 

In order to determine the association between the volumetric measurements and clinical parameters and the risk of NICH, we used the univariable logistic regression analysis. Then the selected clinical and volumetric predictors (determined as *p* < 0.2 in univariable logistic regression analysis) were used to undergo multivariable logistic regression analysis by using the likelihood ratio test with Akaike’s information criterion (AIC) and select the correlated factors by stopping rule. The minimum AIC was taken as indicating the optimal combination of factors. Finally, a model for predicting the risk of NICH based on clinical parameters and volumetric measurements was constructed. A nomogram was drawn to demonstrate the predictive model graphically. It provided the clinician with a quantitative tool to predict the individual probability of ICH risk in neonates. 

### 2.6. Model Assessment

Discrimination ability, calibration ability, and clinical utility are the 3 main aspects that reflect the performance of a predictive model. The receiver operating characteristic (ROC) curve was used to assess the discrimination performance of the developed model. The area under the curve (AUC) was calculated as the quantitative indicator of discrimination ability. The optimal sensitivity, specificity, and accuracy, as well as the cutoff point based on the maximum Youden index were also calculated. 

Calibration ability reflects the consistency between the predicted probability and the actual probability. A calibration curve of the developed model was derived by plotting the observed probabilities against the model-predicted probabilities, using a four-fold dividing strategy based on quartiles. 

We used the decision curve analysis (DCA) to assess the clinical application value of our developed model. Specifically, a DCA curve was plotted to quantify the net benefits for a range of threshold probabilities [12,13], since good accuracy does not necessarily imply that patients would benefit largely from the application of the model.

### 2.7. Statistical Analysis

In our study, two-tailed independent-sample *t*-tests and chi-square tests were used to compare between-group quantitative and qualitative variables (demographic and clinical data). The opening pressure for lumbar puncture, cerebral volume, and CSF volume were expressed as the means ± standard deviation (SD). The statistical tests were two-sided, with statistical significance indicated by *p* < 0.05. All statistical analyses and model building in this study were performed using R software version 3.5.1 (http://www.Rproject.org, accessed on 5 May 2020). Nomogram construction and calibration plots were performed using the “rms” package of the R software version 3.5.1. 

## 3. Results

### 3.1. Clinical Characteristics and Volumetric Segmentation

**Figure 1:**
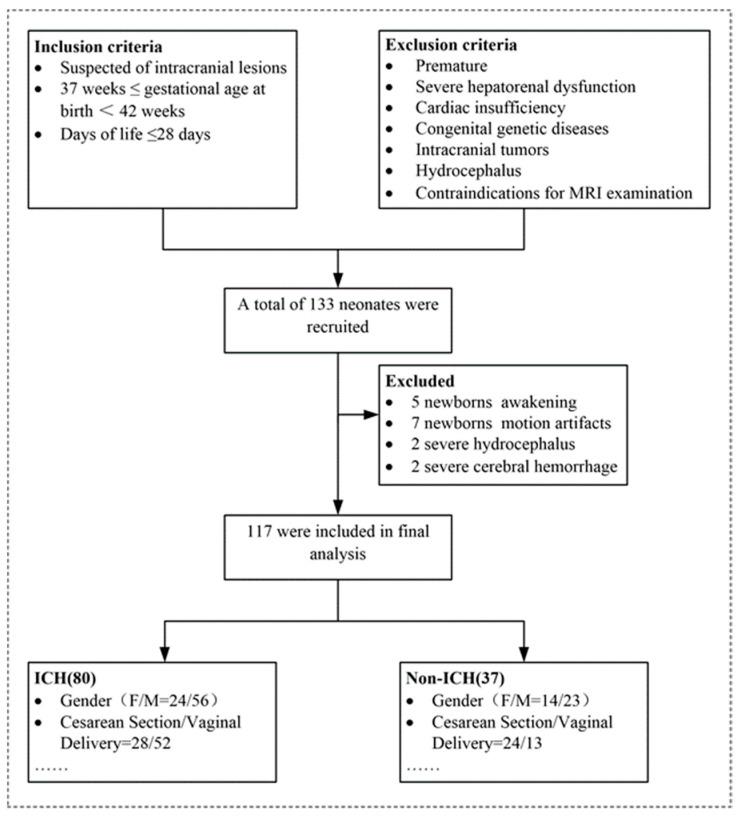
Flowchart demonstrating the inclusion criteria, exclusion criteria, and the patient recruitment process in the present study. Note: ICH = intracranial hypertension.

A total of 133 neonates were recruited in our study, while 16 newborns were excluded due to being awake, motion artifacts, severe hydrocephalus, or severe cerebral hemorrhage. Thus, finally, 117 participants (37 in the non-ICH group and 80 in the ICH group) were included (Figure 1). Statistical analysis revealed that there were no significant differences in sex distribution, gestational age, postmenstrual age, head circumference, Apgar scores, or the mode of delivery between the ICH group and the non-ICH group (Table 1). There was a significant difference in the birth weight between the two groups. Lumbar puncture showed that ICP ranged from 35 to 180 mm H_2_O (mean ICP [SD]:96.94 [30.02] mm H_2_O). Both GM volume (r = 0.22, *p* = 0.02) and total intracranial volume (TICV) (r = 0.18, *p* = 0.04) demonstrated a significant positive correlation with ICP. ICP did not correlate significantly with cerebral WM volume (r = 0.00, *p* = 0.97) or CSF volume (r = −0.00, *p* = 0.95). The TICV (t = −2.40, *p* = 0.02 bilateral, 95% CI (−0.04, −0.00)) and GM volume (t = −3.28, *p* = 0.00 bilateral, 95% CI [−0.04, −0.01]) differed significantly between the ICH group and the non-ICH group. The WM and CSF volumes were not significantly different between the two groups (Table 1). 

### 3.2. Selected Predictors 

**Figure 2:**
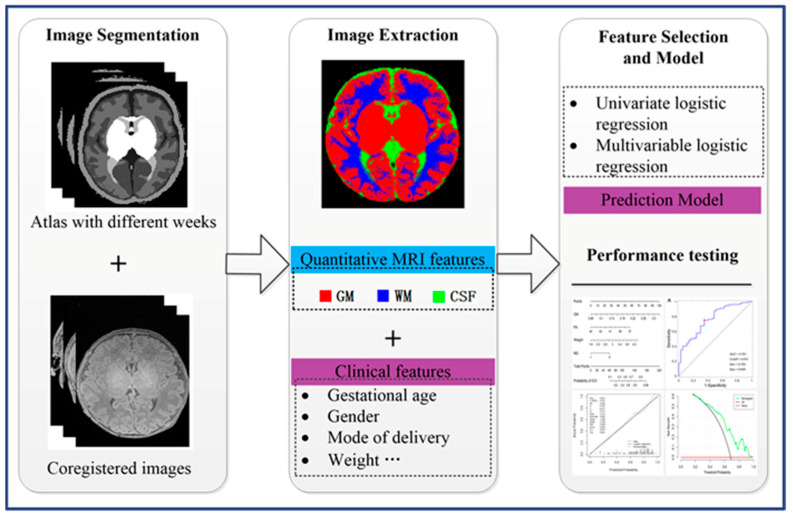
Workflow of the image-based analysis in this study. Note: GM = gray matter, WM = white matter, CSF = cerebrospinal fluid.

**Figure 3:**
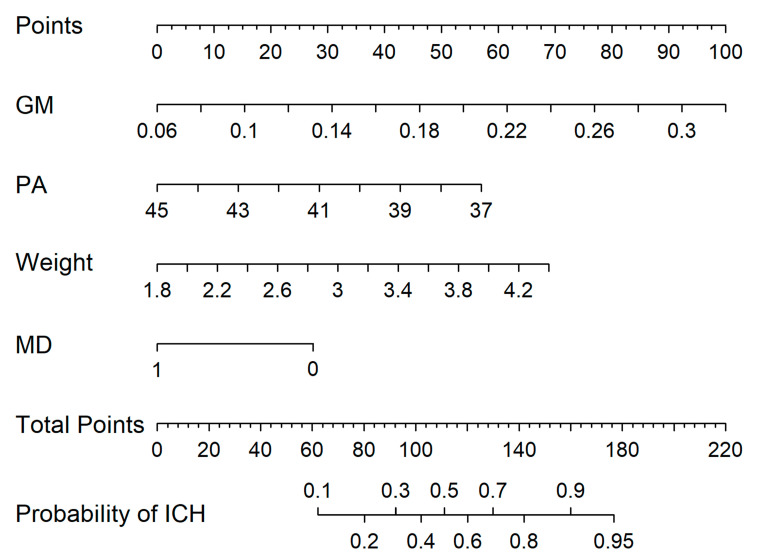
Developed nomogram. The nomogram was developed using the gray matter (GM, liter), postmenstrual age (PA, week), birth weight (kilogram), and mode of delivery (MD,1 means cesarean section and 0 means vaginal delivery).

Using univariable and multivariable logistic regression analysis based on the whole dataset of 117 neonates, 4 (birth weight, mode of delivery, GM, and postmenstrual age) of 11 potential variables were screened out. Then, the remaining variables were included in the multivariable logistic regression analysis as independent predictors (Figure 2). The predictive model was then developed and presented as a nomogram (Figure 3). The nomogram showed that vaginal delivery, a younger gestational age, heavier weight, and greater gray matter volume resulted in a greater risk of NICH.

### 3.3. Performance of the Prediction Model

#### Discrimination and Calibration

**Figure 4:**
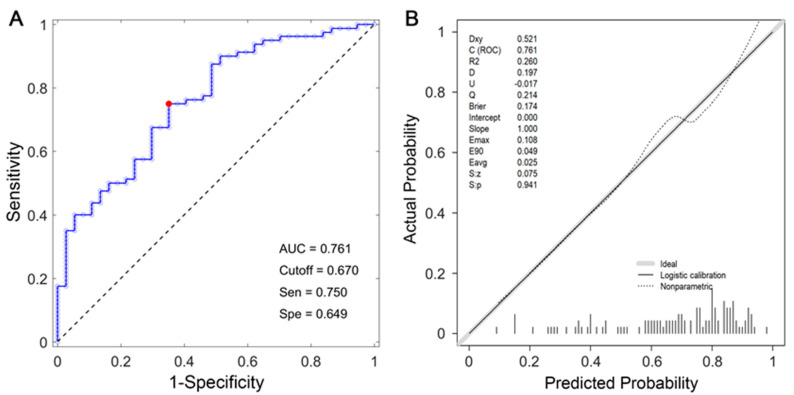
Receiver operating characteristic curve (**A**) and calibration curve (**B**) for the model. The area under the curve was 0.761. The red dot represents the specificity (0.649) and sensitivity (0.750) corresponding to cutoff value (0.670).

Figure 4A shows the ROC curve of the nomogram. The AUC of this prediction model was 0.761. The cutoff point was 0.670 at the maximum Youden index. The sensitivity, specificity, and accuracy at the cutoff point were 64.9%, 75%, and 71.8%, respectively. The calibration curve of the model demonstrated good agreement between the predicted and the observed ICH rates (Figure 4B). The Hosmer-Lemeshow test demonstrated no statistically significant difference between the calibration curve and perfect fit (*p* > 0.05), which suggested that the model neither overestimated nor underestimated the ICH probability of the newborns.

### 3.4. Clinical Utility

**Figure 5:**
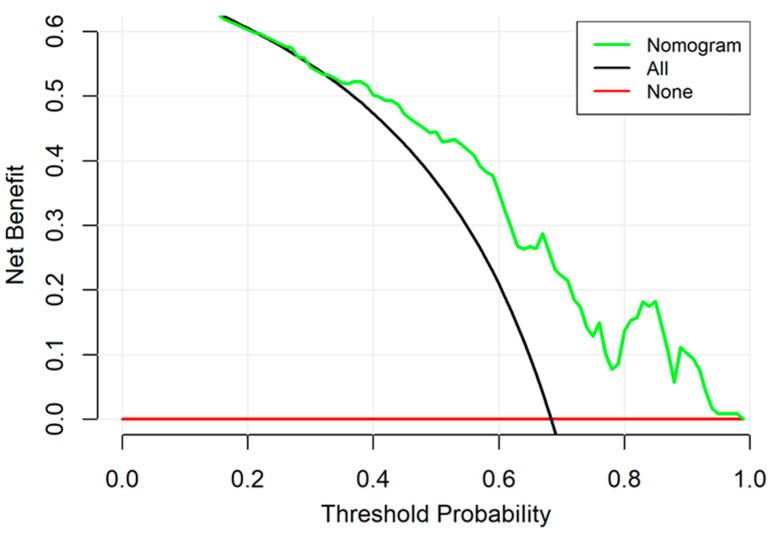
Decision curve analysis for the nomogram. The *y*-axis measures the net benefit. The green line represents the nomogram. The green line represents the nomogram. The red line represents the assumption that no patients have a risk for neonatal intracranial hypertension (NICH) and the black line represents all patients who will develop NICH.

The decision curve for the developed model is presented in Figure 5. The DCA demonstrated that when the threshold probability (Pt) for a neonate with suspected ICH ranged from 0% to 100%, using the model to predict ICH probability yielded more benefit than either the “treat all” or the “treat none” scheme. The added benefits indicated reasonable clinical application utility of the predictive model.

## 4. Discussion

Our study aimed to identify variables that were related to NICH and to construct a nomogram using these variables to predict the risk of NICH. All these variables were either clinical features that are easily accessible or were volumetric data derived from quantitative MRI. After multivariable logistic regression analysis, postmenstrual age, birth weight, mode of delivery, and GM volume were selected as the predictive factors for NICH, with an AUC of 0.761.

Intracranial hypertension is a clinical syndrome caused by various reasons related to increased intracranial pressure. It reflects the changes in intracranial volume and the ability to accommodate additional volume [14]. Generally, ICP > 80 mm H_2_O in neonates is considered to reflect NICH. If the NICH is untreated, it can lead to severely adverse neurological outcomes, poor quality of life, and even increased mortality [11]. Previous studies found that reducing ICP may increase the survival rate among patients with bacterial meningitis [15], which emphasizes the importance of early detection and intervention for ICH. During the neonatal period, Doppler ultrasonography, cerebral blood flow velocity, and near-infrared spectroscopy are common approaches for evaluating ICP [16,17,18,19]. However, the accuracy of these approaches remains problematic, and thus these methods have little validation for clinical use. Kampondeni [20] et al. used noninvasive magnetic resonance imaging to detect cerebral edema and predict the prognosis of pediatric cerebral malaria. In their study, brain volume was obtained using an image-based visual score by radiologists. It is obviously subjective, which depends on the radiologist’s experience in the identification of cerebral edema. However, our study innovatively used a more advanced quantitative magnetic resonance imaging technique. The image was segmented to accurately calculate the volume of brain tissue. Therefore, the results obtained are objective, reliable, and are not affected by the experience of the radiologist. In addition, the nomogram we developed in this study obtained a higher AUC (0.760) than Kampondeni’s study (0.69) and showed satisfactory discrimination and good clinical utility, according to DCA.

Our study showed that vaginal delivery, a younger gestational age, heavier weight, and greater gray matter volume resulted in a greater risk of NICH. The neonatal GM volume was positively correlated to ICP (r = 0.216, *p* = 0.02). The underlying mechanism may be related to cerebral edema, since the GM is the most susceptible brain parenchymal tissue due to its intrinsic physiological structure [21,22]. Cerebral edema is thought to be the underlying pathological basis of NICH in non-traumatic brain injuries, such as hypoxic-ischemic encephalopathy, intracranial infection, severe hyperbilirubinemia, and hypoglycemia [23,24], which were also the main etiologies in the NICH participants in our study. In the ICH group, the frequency of antibiotic use was increased as compared to the non-ICH group, which indicated infections in these populations. Group B Streptococcus commonly colonizes the lower gastrointestinal and genital tracts. It is the main cause of bacterial infection in newborns confirmed through culture in the United States, resulting in significant mortality [25]. During vaginal delivery, the heavier the birth weight, the higher risk of prolonged labor, dystocia, asphyxia, and infection [26]. During vaginal delivery, heavier birth weight may prolong labor and increase the risk of asphyxia and infection of the baby [27]. In addition, Balcer et al. found that obesity in children correlated with an increased risk of primary ICH [28]. 

No previous study has reported a nomogram providing a clinical predictive model for NICH. Our model demonstrated acceptable discrimination performance, with an AUC of 0.761. The optimum sensitivity was 64.9% and specificity was 75%. In a previous study by Ballestero et al., using ICP waveform analysis to predict ICH [29] yielded a sensitivity and specificity of 80% and 100%, respectively. In another study that used optic nerve sheath diameter to differentiate ICH from non-ICH, 93.2% sensitivity and 74% specificity were obtained [30]. The sample size in Ballestero’s study was smaller, and more importantly, the participants in those studies were pediatric subjects, not neonates. There were no previous reports on neonates with which to compare our findings. 

The calibration performance of our developed model was satisfactory. The calibration curve was very close to the ideal line, indicating that our model neither overestimated nor underestimated the probability of NICH in individual patients [31]. Nevertheless, the DCA displayed that our model holds promise for clinical application when the probability threshold was set between 0.4 and 0.9. The net benefits were up to 50%, indicating that, after the deduction of the hazard due to incorrect prediction, there was still a considerable portion of the newborn population that would benefit from the implementation of this model. For DCA, there was no similar model to use for comparison; however, the net benefits of other clinical prediction models have usually not exceeded 30% [32,33], implying that our model was adequate in terms of clinical practice.

### Limitations and Further Study

Some limitations of our study should be noted. First, our sample size was relatively small, especially in the non-ICH group. This is because those who underwent MRIs were usually suspected of having ICH, which tends to reduce the probability of non-ICH. Second, the segmentation of the newborn brain MR images poses additional challenges as compared to that of adults due to motion artifacts, reduced contrast, and increased noise in images, as well as inverted contrast between GM and WM [34,35,36]. Finally and most importantly, our model has not been externally validated yet due to the limited dataset. Further studies to collect larger cohorts of subjects are needed to validate the performance of our developed model.

## 5. Conclusions

In conclusion, in this study, we developed a nomogram to predict the risk of NICH based on quantitative MRI data and clinical parameters. It provided an individualized prediction of neonatal intracranial hypertension risk and facilitated decision making guidance for the early diagnosis and treatment of neonatal intracranial hypertension. External validation from studies using a larger sample size before implementation in the clinical decision-making processes is needed.

## Figures and Tables

**Table 1:** Demographic and clinical findings and segmentation results in neonates with and without ICH.

Characteristic	ICH Group (*n* = 80)	Non-ICH Group (*n* = 37)	*p* Values
Sex	NA	NA	NA
Male, NO. (%)	56 (70)	23 (62)	0.40
Female, NO. (%)	24 (30)	14 (38)	
Gestational age, mean (SD), w	39.38 ± 0.95	39.13 ± 1.07	0.20
Postmenstrual age, mean (SD), w	40.88 ± 1.24	40.90 ± 1.85	0.95
Head Circumference, mean (SD), cm	34.60 ± 1.19	34.43 ± 3.68	0.72
Weight, mean (SD), kg	3.38 ± 0.44	3.10 ± 0.52	0.00
Apgar score, mean (SD)	9.14 ± 1.85	8.70 ± 1.64	0.22
Mode of Delivery	NA	NA	NA
Cesarean Section, NO. (%)	28 (35)	20 (54)	0.51
Vaginal Delivery, NO. (%)	52 (65)	17 (46)	
TICV, mL	468.85 ± 48.21	444.94 ± 54.23	0.02
GM, mL	190.55 ± 42.55	163.51 ± 38.90	0.00
WM, mL	167.27 ± 36.59	172.74 ± 43.89	0.48
CSF, mL	71.17 ± 21.76	70.72 ± 19.48	0.92

Note: Data are the mean ± standard deviation or number (%). ICH = Intracranial hypertension, TICV = Total intracranial volume, GM = gray matter, WM = white matter, CSF = cerebral spinal fluid. NA = not applicable, CI = confidence interval.

## Data Availability

Data is unavailable due to privacy and ethical restrictions.
